# Reg-2, A Downstream Signaling Protein in the Ciliary Neurotrophic Factor Survival Pathway, Alleviates Experimental Autoimmune Encephalomyelitis

**DOI:** 10.3389/fnana.2016.00050

**Published:** 2016-05-09

**Authors:** Hong Jiang, Ke-Wei Tian, Fan Zhang, Beibei Wang, Shu Han

**Affiliations:** ^1^Department of Electrophysiology, Sir Run Run Shaw Hospital, Medical College, Zhejiang UniversityHangzhou, China; ^2^Institute of Anatomy and Cell Biology, Medical College, Zhejiang UniversityHangzhou, China; ^3^Core Facilities, Zhejiang University School of MedicineHangzhou, China

**Keywords:** CNTF, experimental autoimmune encephalitis, multiple sclerosis, neuronal protection, Reg-2

## Abstract

Ciliary neurotrophic factor (CNTF), originally described as a neurocytokine that could support the survival of neurons, has been recently found to alleviate demyelination, prevent axon loss, and improve functional recovery in a rat model of acute experimental autoimmune encephalomyelitis (EAE). However, poor penetration into the brain parenchyma and unfavorable side effects limit the utility of CNTF. Here, we evaluated the therapeutic potential of a protein downstream of CNTF, regeneration gene protein 2 (Reg-2). Using multiple morphological, molecular biology, and electrophysiological methods to assess neuroinflammation, axonal loss, demyelination, and functional impairment, we observed that Reg-2 and CNTF exert similar effects in the acute phase of EAE. Both treatments attenuated axonal loss and demyelination, improved neuronal survival, and produced functional improvement. With a smaller molecular weight and improved penetration into the brain parenchyma, Reg-2 may be a useful substitute for CNTF therapy in EAE and multiple sclerosis (MS).

## Introduction

Multiple sclerosis (MS) is a neurodegenerative disease of the central nervous system (CNS) that is characterized by CNS inflammation, demyelination, axonal degeneration, neuron loss, and neurological deficits (Bannerman et al., 2005). The corresponding animal model, experimental autoimmune encephalomyelitis (EAE), is an experimental paradigm that is widely used for the study of MS (Hou et al., 2012).

A previous study showed that treatment with ciliary neurotrophic factor (CNTF) robustly promoted myelin formation, reduced perivascular T cell infiltration, and limited the extent of microglial activation in EAE (Kuhlmann et al., 2006). This study of CNTF also reported significantly higher numbers of oligodendrocytes, axons, and neurons in EAE mice that had been treated with high doses of CNTF. However, CNTF is a relatively large molecule (molecular mass: 25 kDa; Webster, 1997) and does not easily cross the blood-brain barrier (BBB). Previous primate and rodent studies have also suggested that CNTF needs to be delivered directly to the parenchyma of the striatum to be effective (Emerich, 2004). Moreover, the adverse effects of CNTF treatment, such as fever, malaise, and weight loss, limit its potential clinical use (Kuhlmann et al., 2006).

Regeneration gene protein 2 (Reg-2) is a relatively small (molecular mass: 16 kDa) secreted protein that is expressed in motoneurons during development and induced by CNTF-related cytokines in the adult CNS (Nishimune et al., 2000). Reg-2 has been shown to facilitate the regeneration of motor and sensory neurons after peripheral nerve injury in the adult rat, and to act as an essential intermediate neurotrophic factor in the pathways through which CNTF supports motor neuron survival during development (Averill et al., 2002). We recently demonstrated the neuroprotective effects of Reg-2 in a rodent model of spinal cord injury (Fang et al., 2010, 2011). Moreover, in preliminary experiments, we investigated the adverse effects of intravenously delivered Reg-2 naïve rats and did not observe fever, lackluster hair or hair loss, haggard appearance, or weight loss (data not shown). However, whether Reg-2 can be used as a suitable substitute for CNTF in the rodent model of EAE requires confirmation.

In this study, we first analyzed the permeability of the BBB to Reg-2 and CNTF, and then delivered Reg-2 or CNTF directly into the lateral ventricle of EAE rats to avoid confounding effects due to differences in CNS penetration. We subsequently assessed the degree of inflammation, axonal loss, demyelination, and functional impairment in treated EAE rats.

## Materials and Methods

### Animals

Fifteen adult male Sprague-Dawley rats and 56 adult male Lewis rats weighing 250–300 g were obtained from the Laboratory Animal Services Centre of Zhejiang University. Rats were housed in temperature- and humidity-controlled chambers and had access to food and water *ad libitum*. All animal procedures used in this study were carried out in accordance with the National Institutes of Health Guide for the Care and Use of Laboratory Animals, and with approval from the Animal Ethics Committee of Zhejiang University.

### Analysis of the Permeability of the Blood-Brain Barrier to Reg-2 and CNTF

Recombinant Reg-2 (16 kDa) and CNTF (25 kDa) labeled with ^13^C6-Leucine were purchased from GL Biochem Ltd. (Shanghai, China). Ten Sprague-Dawley rats were divided randomly into two groups and anesthetized using 40 mg/kg pentobarbital. Subsequently, a bolus (1 mL) containing ^13^C6-labeled Reg-2 or CNTF in 0.9% saline (1 mg/mL) was rapidly injected into the brachial vein. Rats were sacrificed 1 h after injection, the brain was collected, and the choroid plexus and meninges were carefully removed. Tissues were then lyophilized and dry weights were determined using a microbalance (Poduslo and Curran, 1996). Tissues were lysed in a solution containing 1% Triton X-100 and a protease inhibitor cocktail. Protein mixtures were then separated on an SDS-PAGE gradient gel (7.5–15%), stained with Coomassie blue, and the bands of the correct molecular weight proteins were identified (Zhang et al., 2012). Liquid chromatography-mass spectrometric (LC-MS) analysis was used to monitor the amount of the ^13^C6-leucine labeling in each group. Scans of the entire LC-MS/MS data set were analyzed using Qual Browser Xtract Software (Thermo Scientific, Bremen, Germany; Romijn et al., 2005). Values from five rats in the control group, which received only 0.9% saline, were used for subtracting background values.

### Animals and EAE Induction

Of 56 Lewis rats, eight were used as normal controls and the remaining 48 were randomly divided into three groups: a vehicle (saline)-treated group, a Reg-2-treated group, and a CNTF-treated group. Reg-2 was synthesized by Shanghai Science Peptide Biological Technology Co., Ltd (Shanghai, China) and CNTF was obtained from PeproTech Inc. (Rocky Hill, NJ, USA).

EAE was induced prior to the initiation of treatments with a subcutaneous injection of 0.2 mL of a 1:1 mixture of guinea pig spinal cord homogenate (GPSCH) and complete Freund’s adjuvant (CFA) containing 0.5 mg of heat-killed *Mycobacterium tuberculosis* (Difco Laboratories, Detroit, MI, USA). Rats in the normal control group were injected with CFA emulsified 1:1 with 0.9% saline. Immediately thereafter and again 48 h later, each rat received an intraperitoneal injection of 300 ng Pertussis toxin in 0.1 mL sterile water (Sigma-Aldrich, St. Louis, MO, USA; Ma et al., 2010; Yin et al., 2010).

Animals were assessed for clinical signs of disease by blinded observers on a daily basis. Assessments were initiated immediately following EAE induction on day 0 and continued until the time of sacrifice. Disease severity was graded using a 0–5 scale: grade 0 = no signs of paralysis, grade 1 = partial loss of tail tonicity, grade 2 = complete loss of tail tonicity, grade 3 = unsteady gait and mild paralysis, grade 4 = hind limb paralysis and incontinence, and grade 5 = moribund or death (Yu et al., 2010).

### Preparation of Osmotic Pumps for Drug Delivery

CNTF, Reg-2, or vehicle was delivered continuously over a 2-week period via implanted Alzet osmotic minipumps (pump model 2002; Alzet, Billerica, MA, USA; 1 mg/ml, 0.5 μL/h). Pumps were prepared under sterile conditions, filled with 0.9% saline (for the vehicle-treated group: *n* = 16), or Reg-2 or CNTF in saline (1 mg/ml, *n* = 16 per group; Cui et al., 2004; Fang et al., 2011), and primed overnight at room temperature. All pumps were implanted immediately after EAE induction. The drug delivery process has been previously described (Fang et al., 2013).

### Neurophysiological Examination

Cortical somatosensory evoked potentials (c-SEPs) and cortical motor evoked potentials (c-MEPs) were recorded at 2 weeks (peak EAE severity in vehicle-treated rats) and 8 weeks (recovery stage in vehicle-treated rats) post-immunization in five rats from each group immediately prior to sacrifice. Recordings were performed by blinded observers as previously described (c-SEP procedure: Troncoso et al., 2000; Devaux et al., 2003; All et al., 2009; Fang et al., 2013; c-MEP procedure: Bolay et al., 2000; Fang et al., 2013).

### Perfusion and Tissue Processing

Animals were sacrificed at 2 weeks and 8 weeks post-immunization (five animals per time point per group). Half of the brain and spinal cord tissues from each animal were prepared for histological assessment and immunohistochemical and immunofluorescence staining, and the remaining tissues were examined using transmission electron microscopy. Perfusion and tissue processing were performed as previously described (Han et al., 2013; Zhang et al., 2014).

### Histological Assessment

Digital photomicrographs were obtained at 200× magnification in three visual fields per section. Cresyl violet (Nissl) staining was used to assess inflammation and neuronal survival. Inflammatory cell infiltration was assessed according to the following scale (Romijn et al., 2005): 0 = no inflammation, 1 = cellular infiltration proximal to the blood vessels and meninges, 2 = mild cellular infiltration into the parenchyma (1–10 cells/section), 3 = moderate cellular infiltration into the parenchyma (11–100 cells/section), and 4 = severe cellular infiltration into the parenchyma (100+ cells/section). For neuronal counts, large multipolar spinal cord anterior horn motoneurons and pyramid-shaped precentral gyrus motoneurons were counted if the cells displayed a well-defined nucleolus and a cell body with adequate amounts of endoplasmic reticulum.

Immunohistochemical staining of myelin basic protein (MBP) was used to evaluate the degree of demyelination. Demyelination was scored as follows (Yin et al., 2010): 0 = normal white matter, 1 = rare foci of demyelination, 2 = few areas of demyelination, 3 = confluent perivascular or subpial demyelination, 4 = massive perivascular and subpial demyelination involving one half of the spinal cord, with the presence of cellular infiltrates into the CNS parenchyma, and 5 = extensive perivascular and subpial demyelination involving the whole cord section, with the presence of cellular infiltrates into the CNS parenchyma.

Bielschowsky’s silver staining was performed to estimate axonal loss (Bolay et al., 2000; Han et al., 2013) and assessed according to the following scale (Yin et al., 2010): 0 = no loss, 1 = few foci of superficial loss involving less than 25% of tissue, 2 = foci of deep axonal loss involving greater than 25% of tissue, and 3 = diffuse and widespread axonal loss.

### Immunohistochemical and Immunofluorescence Staining

Immunohistochemical staining with anti-CD4 (1:500, Abcam, Cambridge, MA, USA), anti-COX-2 (COX-2; 1:1000, BioVision, Milpitas, CA, USA), anti-activated caspase-3 (1:500; Cayman Chemical, Ann Arbor, MI, USA), anti-NF-κB p65 (1:500, Abcam), and anti-MBP (1:500, Abcam), and immunofluorescence staining with polyclonal anti-BCL-2 (1:200; Santa Cruz, Dallas, TX, USA), anti-TNF-α (1:1000; ProSci Inc., San Diego, CA, USA), anti-activated BAX (1:200; Santa Cruz), anti-GAP43, and anti-CD68/ED1 (1:100; Santa Cruz) were performed as previously described (Cui et al., 2004). Double-immunostaining was performed using anti-CD4 (1:500, Abcam, Cambridge, MA, USA, green) and anti-CCR3 (1:500, Abcam, Cambridge, MA, USA) to identify Th2-polarized cells. Primary antibody omission controls were performed as negative controls.

Five sections from the motor cortex and bilateral anterior horns of the spinal cord for each animal were randomly selected and images were photographed under 20× magnification in three vision fields per section. The numbers of cells positive for COX-2-, NF-κB p65, GAP43, BAX, BCL-2 and caspase-3 were counted (cells positive for GAP43, BAX, and BCL-2 were counted via immunofluorescence staining and cells positive for COX-2-, NF-κB p65, and caspase-3 were counted using a counter-staining method).

### Electron Microscopy

Processing for electron microscopy was performed as previously described (Bolay et al., 2000; Han et al., 2013). Images were captured in different regions of the motor cortex and lumbar spinal cord. All histological assessments were performed in a blinded fashion.

### Quantification of Cytokine Levels by Enzyme-Linked Immunosorbent Assay (ELISA)

Peripheral blood samples were collected from rats sacrificed by decapitation at 2 weeks and 8 weeks post-immunization (*n* = 3 per time point per group). ELISAs for IL-2 (Abcam), IL-4 (Abcam), TGF-β (R&D Systems), and IFN-γ (BioLegend Inc.) were performed according to manufacturer specifications (Bolay et al., 2000; Han et al., 2013). Optical density was measured at 450 nm and analyzed using GraphPad Prism 4 (GraphPad Software, Inc., San Diego, CA, USA).

### Western Blotting

Rats were sacrificed by decapitation at 2 weeks and 8 weeks post-immunization (*n* = 3 per time point per group). Whole brain cortical tissue and 10 mm lumbar spinal cord segments were prepared for western blotting. Processing was performed as previously described (Bolay et al., 2000; Han et al., 2013). Primary antibody omission controls were performed as negative controls.

### Statistical Analysis

Differences in protein expression were analyzed using a 2-way ANOVA followed by *post hoc* Tukey *t*-tests. The Kruskal-Wallis nonparametric analysis was used for data presented as percentages. Differences in clinical and histological scores were analyzed using the Mann-Whitney *U*-test. All analyses were performed using SPSS 13.0 software unless otherwise specified. *P*-values less than 0.05 were considered to be statistically significant. All statistical graphs were generated with GraphPad Prism Version 4.0 (GraphPad Prism Software, Inc., San Diego, CA, USA).

## Results

### Reg-2 has Greater BBB Permeability than CNTF

An LC-MS-MS analysis revealed that the mean ^13^C abundance in normal control brain tissue was 1.09%, and this finding was consistent with a previous report (Ross et al., 2003). Thirty minutes after intravenous injection of CNTF or Reg-2, the mean ^13^C abundance in brain parenchyma was 1.15% in the ^13^C-CNTF group and 1.26% in the ^13^C-Reg-2 group; the latter value was significantly higher than the former (*P* < 0.05).

### Both CNTF Treatment and Reg-2 Treatment Reduce the Severity of EAE

c-SEP and c-MEP latencies were used as surrogates of conduction speed, and amplitudes indicated the number of surviving fibers. EAE induction prolonged the latencies to waveform initiation and decreased the peak amplitudes of both c-SEPs (Tables 1, 2) and c-MEPs (Tables 3, 4) in EAE rats. Both CNTF treatment and Reg-2 treatment significantly attenuated disease-associated delays in latency and decreases in amplitude. Furthermore, there was no difference between CNTF treatment and Reg-2 treatment in terms of these effects (*P* > 0.05).

**Table 1:** **CNTF treatment and Reg-2 treatment reduce c-SEP latency and increase c-SEP amplitude at 2 weeks post-immunization**.

2 weeks			
	Latency (ms)	
Groups	*N*	*P*	Wave amplitude (μV mean ± SD)
Normal	15.53 ± 1.48**	20.45 ± 2.33**	1.22 ± 0.25**
EAE	26.91 ± 3.32	35.73 ± 3.54	0.39 ± 0.03
CNTF	21.35 ± 1.17*	27.1 ± 0.77*	0.72 ± 0.16*
Reg-2	18.2 ± 0.71*	24.78 ± 0.77*	0.65 ± 0.19*

*P < 0.05 vs. vehicle-treated EAE rats. **P < 0.01 vs. vehicle-treated EAE rats. N, negative deflection; P, positive deflection.

**Table 2:** **CNTF treatment and Reg-2 treatment reduce c-SEP latency and increase c-SEP amplitude at 8 weeks post-immunization**.

8 weeks			
	Latency (ms)	
Groups	*N*	*P*	Wave amplitude (μV mean ± SD)
Normal	13.78 ± 1.90**	19.23 ± 1.64**	2.60 ± 0.24**
EAE	25.58 ± 6.36	37.13 ± 1.10	0.58 ± 0.08
CNTF	18.95 ± 4.57*	26.13 ± 1.15*	0.90 ± 0.26*
Reg-2	15.97 ± 0.12*	21.53 ± 0.21*	1.30 ± 0.40**

*P < 0.05 vs. vehicle-treated EAE rats. **P < 0.01 vs. vehicle-treated EAE rats. N, negative deflection; P, positive deflection.

**Table 3:** **CNTF treatment and Reg-2 treatment reduce c-MEP latency and increase c-MEP amplitude at 2 weeks post-immunization**.

2 weeks		
Groups	Latency (ms)	Wave amplitude (μV mean ± SD)
Normal	5.15 ± 0.17*	3.02 ± 0.73**
EAE	7.73 ± 2.31	0.21 ± 0.01
CNTF	5.8 ± 0.25*	2.13 ± 0.23**
Reg-2	5.57 ± 0.26*	2.03 ± 0.38**

*P < 0.05 vs. vehicle-treated EAE rats. **P < 0.01 vs. vehicle-treated EAE rats.

**Table 4:** **CNTF treatment and Reg-2 treatment reduce c-MEP latency and increase c-MEP amplitude at 8 weeks post-immunization**.

8 weeks		
Groups	Latency (ms)	Wave amplitude (μV mean ± SD)
Normal	3.56 ± 0.77**	3.78 ± 1.06**
EAE	6.8 ± 0.15	0.22 ± 0.08
CNTF	5.3 ± 0.08*	1.2 ± 0.23**
Reg-2	5.2 ± 0.14*	1.63 ± 0.15**

*P < 0.05 vs. vehicle-treated EAE rats. **P < 0.01 vs. vehicle-treated EAE rats.

**Figure 1:**
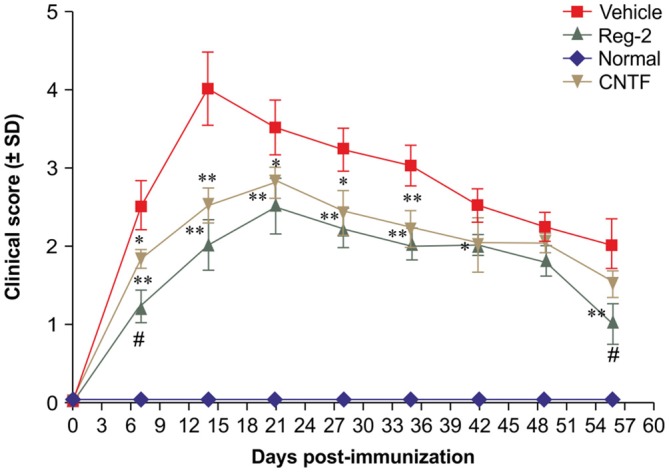
**Ciliary neurotrophic factor (CNTF) treatment and regeneration gene protein 2 (Reg-2) treatment similarly improve the clinical progression of experimental autoimmune encephalomyelitis (EAE).** The clinical progression of EAE was attenuated in rodents treated with CNTF or Reg-2, as measured by disease scoring. *P < 0.05 vs. vehicle-treated group at the same time point. **P < 0.01 vs. vehicle-treated group at the same time point. A delay in the onset of EAE symptoms and a postponed peak stage were observed in CNTF- and Reg-2-treated rats. During the onset and recovery stages, clinical scores were lower in Reg-2-treated rats than in CNTF-treated rats (^#^*P* < 0.05 vs. the CNTF-treated group).

Per functional scoring, EAE symptoms appeared in vehicle-treated rats on days 7–9 post-immunization and peaked (average clinical score = 3.5–4.0) at 2 weeks post-immunization. Thereafter, clinical scores gradually declined and acute EAE rats underwent spontaneous recovery by 8 weeks post-immunization (average clinical score = 1.5–2.0). The disease progression of CNTF- and Reg-2-treated rats was similar to that of vehicle-treated rats, but the disease onset was more gradual and the peak disease severity was delayed to 3 weeks post-immunization. Furthermore, clinical scores were significantly lower in CNTF- and Reg-2-treated rats than in vehicle-treated rats at all time points (Figure 1).

### CNTF Treatment and Reg-2 Treatment Limit the Extent of CNS Infiltration and Inflammation in EAE

**Figure 2:**
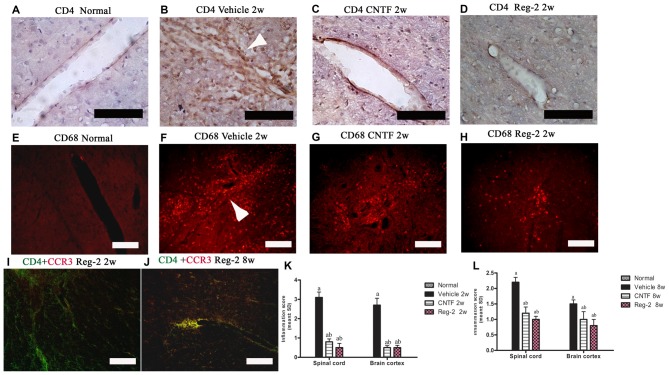
**CNTF treatment and Reg-2 treatment inhibit inflammatory cell infiltration.** At 2 weeks post-immunization, the infiltration of CD4+ T lymphocytes **(A–D)** and CD68^+^ macrophages **(E–H)** was observed proximal to blood vessels and in the central nervous system (CNS) parenchymata of vehicle-treated rats. Both CNTF and Reg-2 treatments alleviated inflammatory cell infiltration. **(A–D)** Immunostaining for CD4 (a marker of extravasated T lymphocytes) and counterstaining with hematoxylin. **(E–H)** Immunofluorescence staining for CD68 (a marker of activated microglia/extravasated macrophages). **(I,J)** Double immunostaining for CD4 (green) and CCR3 (red), a marker for Th2-polarized cells. Scale bar = 100 μm. The arrow shows infiltrating inflammatory cells surrounding blood vessels in a “perivascular cuffing” formation. **(K,L)** CNTF and Reg-2 treatments attenuated CNS inflammation at 2 weeks **(K)** and 8 weeks **(L)** post-immunization, as estimated by inflammation score. (a) *P* < 0.05 vs. vehicle-treated EAE rats; (b) *P* < 0.05 vs. CNTF-treated EAE rats.

To quantify inflammatory cell infiltration into the CNS, we performed immunostaining for CD4, a marker of extravasated T lymphocytes, and CD68, a marker of activated microglia and extravasated macrophages. At the peak stage of acute EAE (2 weeks post-immunization), vehicle-treated rats exhibited a significant increase in the number of infiltrating inflammatory cells. When compared with the normal group (Figures 2A,E), diffuse infiltration was observed not only around the blood vessels, but also throughout the brain and spinal cord parenchymata (Figures 2B,F,G; Supplementary Figure 1C). Less severe perivascular and parenchymal infiltration was observed in CNTF-treated rats (Figures 2C,H; Supplementary Figure 1D) and Reg-2-treated rats (Figures 2D,I; Supplementary Figure 1E) at this time point. In addition, the inflammatory scores of CNTF- and Reg-2-treated rats were also significantly lower than that of vehicle-treated rats at 2 weeks post-immunization (Figure 2K). At 8 weeks post-immunization, inflammatory cell infiltration and inflammatory scores were increased in CNTF- and Reg-2-treated rats (Figure 2L, Supplementary Figures 1G,H), but remained significantly lower than those observed in vehicle-treated rats. (Figure 2L, *P* < 0.05). Moreover, the co-localization of CCR3 (a marker for Th2-polarized cells) and CD4 on infiltrating T cells was visibly increased at this time point relative to 2 weeks post-immunization (Figures 2I,J).

### CNTF Treatment and Reg-2 Treatment Inhibit Demyelination and Improve Remyelination in EAE

**Figure 3:**
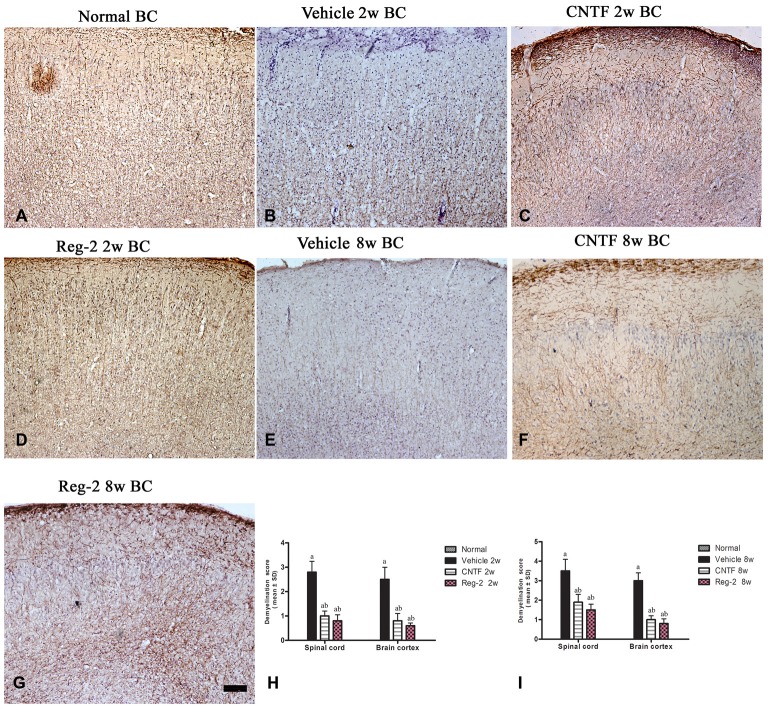
**CNTF treatment and Reg-2 treatment reduce demyelination.** Sections were stained for myelin basic protein (MBP) and counterstained with hematoxylin **(A–G)**. **(H,I)** The effect of CNTF and Reg-2 treatments on myelination at 2 weeks **(H)** and 8 weeks **(I)** post-immunization, as estimated by demyelination score. (a) *P* < 0.05 vs. vehicle-treated EAE rats; (b) *P* < 0.05 vs. CNTF-treated EAE rats. Scale bar = 100 μm.

Immunohistochemical labeling of MBP was used as a surrogate indicator of myelination (Figure 3). Large confluent areas of demyelination were observed in vehicle-treated EAE rats relative to normal rats at 2 weeks and 8 weeks post-immunization (Figures 3A–D). CNTF or Reg-2 treatment attenuated the development of demyelination relative to vehicle-treated rats at these time points (Figure 3).

**Figure 4:**
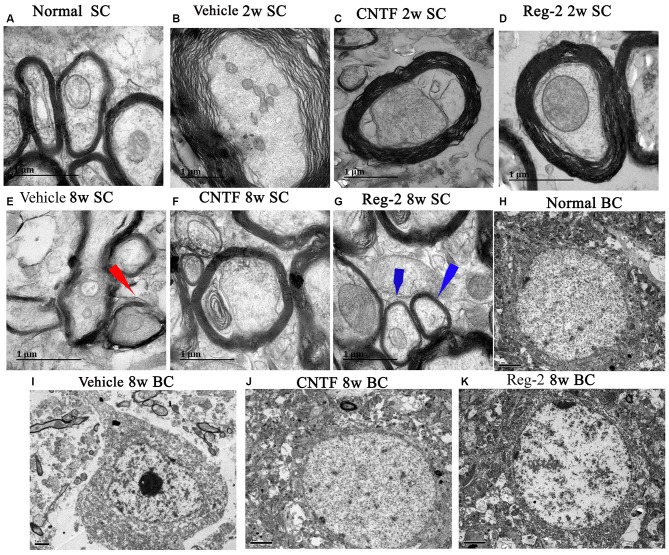
**CNTF treatment and Reg-2 treatment prevent demyelination, axonal loss, and neuronal apoptosis.** Representative electron micrographs at 2 weeks and 8 weeks post-immunization. BC, Brain cortex; SC, Spinal cord. **(A)** Normal myelinated axons exhibiting dark, ring-shaped myelin sheaths. **(B)** Sheath splitting and vacuoles showing loose and fused changes were observed in vehicle-treated rats at 2 weeks post-immunization. In CNTF- **(C)** and Reg-2-treated **(D)** rats at 2 weeks post-immunization, some vacuolated sheaths were observed, but most of the dark ring-shaped myelin sheath structures were retained. **(E)** Myelin lamellae undergoing vesicular disintegration and concurrent remyelination (red arrow) were observed in vehicle-treated rats at 8 weeks post-immunization. In contrast, CNTF- **(F)** and Reg-2-treated **(G)** rats showed a greater number of newly formed myelin sheaths (blue arrow) at 8 weeks post-immunization. **(H)** Normal nuclei of neurons with uncondensed chromatin. **(I)** In vehicle-treated rats, apoptotic neurons with remarkably shrunken nuclei containing condensed, fragmented, and marginalized nuclear chromatin were observed. In CNTF- **(J)** and Reg-2-treated **(K)** rats, the ultrastructure of myelin was retained and appeared relatively normal. **(A–G)** Scale bar = 1 μm; **(H–K)** Scale bar = 2 μm.

Transmission electron microscopy examination further revealed that, at 2 weeks post-immunization in vehicle-treated rats, a considerable amount of the myelin sheath displayed splitting and vacuolar changes when compared with the normal group (Figure 4A); axons were shrunken and covered by disrupted myelin (Figure 4B). In contrast, CNTF- and Reg-2-treated rats exhibited lesser vacuolar changes at 2 weeks post-immunization (Figures 4C,D). At 8 weeks post-immunization, demyelination and remyelination appeared simultaneously in vehicle-treated rats; additionally, nuclei showed signs of apoptosis, including condensed, fragmented, and marginalized nuclear chromatin (Figures 4E,I). A greater number of remyelinated fibers was observed in CNTF- and Reg-2-treated rats (Figures 4F,G); additionally, neighboring nuclei presented a normal ultrastructure (Figures 4J,K) just like the nuclei of normal group (Figure 4H).

### CNTF Treatment and Reg-2 Treatment Prevent Axon Loss

**Figure 5:**
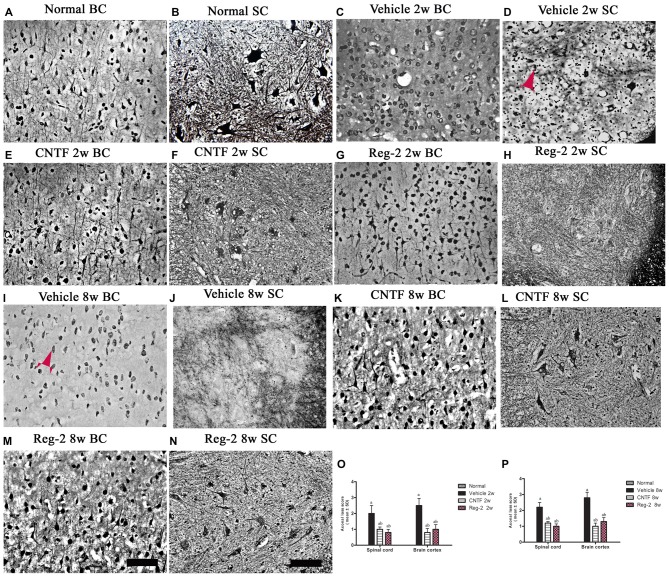
**CNTF treatment and Reg-2 treatment alleviate axonal loss at both 2 weeks and 8 weeks post-immunization. (A,B)** Normal control. **(C,D,I,J)** Numerous axons undergoing gradual loss and exhibiting deformed and ovoid formations in vehicle-treated rats at 2 weeks and 8 weeks post-immunization, indicated by arrows. In CNTF- **(E,F,K,L)** and Reg-2-**(G,H,M,N)** treated rats, more axons were retained relative to the vehicle-treated group at both 2 weeks **(O)** and 8 weeks **(P)** post-immunization, as estimated by axonal loss score. (a) *P* < 0.05 vs. vehicle-treated EAE rats; (b) *P* < 0.05 vs. CNTF-treated EAE rats. Scale bar = 100 μm.

Bielschowsky’s silver staining revealed reductions in CNS axonal density in vehicle-treated rats relative to normal rats at 2 weeks (Figures 5C,D) and 8 weeks (Figures 5I,J) post-immunization. Vehicle-treated rats also exhibited axonal loss and injured axons displaying swelling, deformation, and ovoid formations (arrow in Figure 5D) relative to normal rats (Figures 5A,B) at 2 weeks post-immunization. At 8 weeks post-immunization in the vehicle-treated group, many neurons exhibited almost complete axonal loss, such that only the injured cell bodies were observed (arrows in Figure 5I). A larger number of axons with relatively normal formation were observed in CNTF- and Reg-2-treated rats than in vehicle-treated rats (Figure 5). Axonal loss scores (Figures 5O,P) also confirmed the axon-protective effects of CNTF and Reg-2 treatments. Moreover, there was no difference between the axonal loss scores of CNTF- and Reg-2-treated rats (Figures 5O,P).

### CNTF Treatment and Reg-2 Treatment Suppress the Expression of Pro-Inflammatory Factors and Increase the Expression of Anti-Inflammatory Factors in EAE

**Figure 6:**
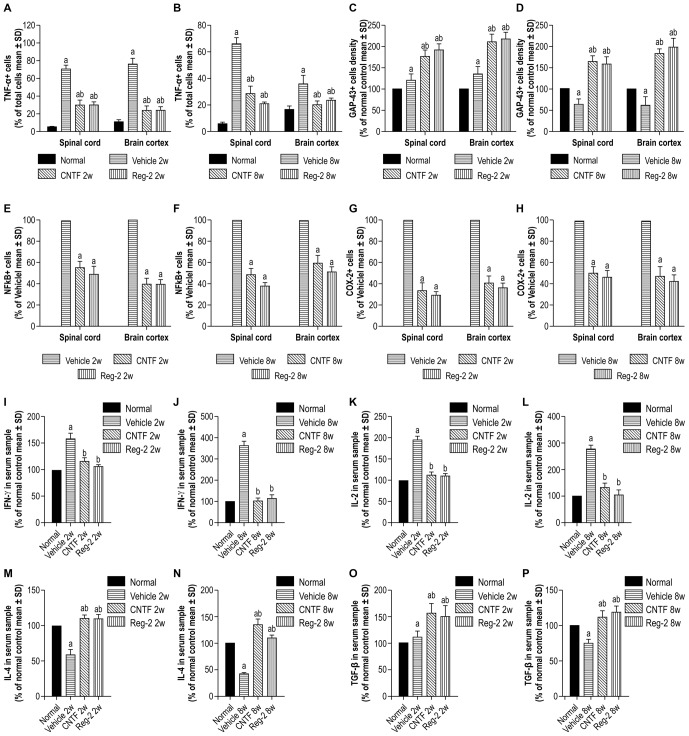
**CNTF treatment and Reg-2 treatment effectively reduce the expression of pro-inflammatory factors.** The pro-inflammatory factors TNF-α **(A,B)**, NF-κB **(E,F)**, COX-2 **(G,H)**, IFN-γ **(I,J)**, and IL-2 **(K,L)** were attenuated while GAP-43 **(C,D)** and the anti-inflammatory cytokines IL-4 **(M,N)** and TGF-β **(O,P)** were increased in CNTF- and Reg-2-treated rats. (a) *P* < 0.05 vs. normal control; (b) *P* < 0.05 vs. vehicle-treated EAE rats. **(E–H)** (a) *P* < 0.05 vs. vehicle-treated EAE rats.

**Figure 7:**
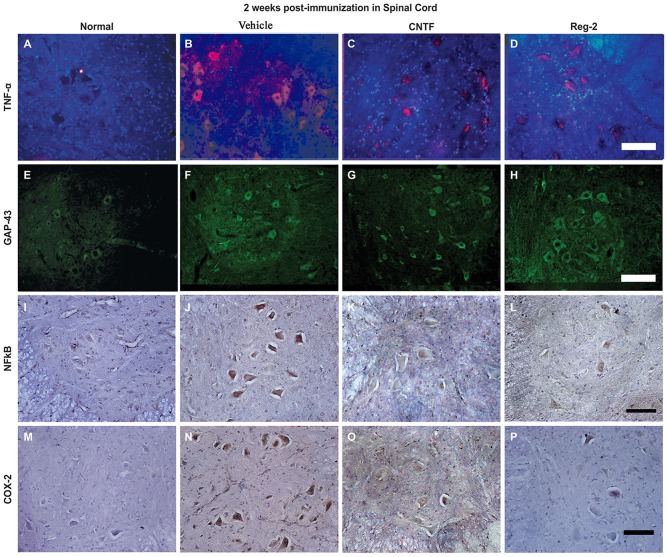
**The expression of pro-inflammatory factors in response to CNTF treatment and Reg-2 treatment.** At 2 weeks post-immunization, the expression of TNF-α (**A–D**, TRIFC-conjugated immunofluorescent staining, red), GAP-43 (**E–H**, FITC-conjugated immunofluorescent staining, green), NF-κB **(I–L)**, and COX-2 (**M–P**, immunohistochemical staining counterstained with hematoxylin) were increased in vehicle-treated rats relative to normal control rats. The expression of pro-inflammatory factors was attenuated while the expression of GAP-43 was enhanced in both CNTF- and Reg-2-treated rats.

COX-2, NF-κB, TNF-α, and GAP-43 were detected in CNS tissues by immunostaining (Figures 6, 7), and TGF-β, IFN-γ, IL-2, and IL-4 were measured in serum by ELISA (Figure 6). The expression of important pro-inflammatory factors such as COX-2 and NF-κB and cytokines such as TNF-α and IL-2 were significantly increased in vehicle-treated EAE rats at 2 weeks and 8 weeks post-immunization. CNTF and Reg-2 treatments markedly reduced the expression of these factors (Figures 6, 7). Conversely, anti-inflammatory cytokines such as IL-4 and TGF-β were clearly downregulated in vehicle-treated EAE rats relative to normal rats at 2 weeks and 8 weeks post-immunization. In contrast, anti-inflammatory cytokines were increased in CNTF- and Reg-2- treated rats (Figure 6).

EAE-induction fractionally increased the expression of GAP-43 in the early stages of disease, but GAP-43 expression decreased as EAE progressed. Of note, CNTF and Reg-2 treatments markedly upregulated the expression of GAP-43 at both 2 weeks and 8 weeks post-immunization (Figures 6C,D, 7E–H). There were no significant differences observed between CNTF- and Reg-2-treated rats (Figures 6, 7).

### CNTF Treatment and Reg-2 Treatment Reduce Apoptosis and Neuronal Loss in EAE

**Figure 8:**
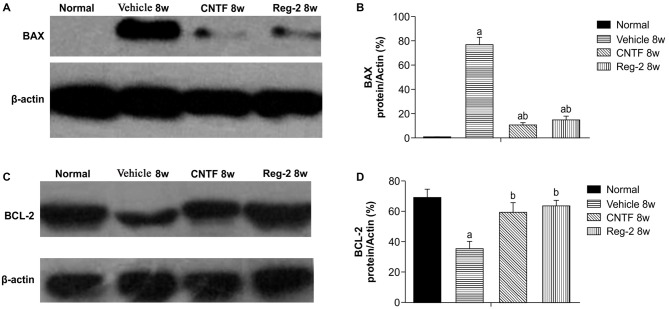
**Effect of CNTF and Reg-2 on apoptosis at 8 weeks post-immunization. (A,C)** In vehicle-treated rats, expression of the pro-apoptotic regulator BAX was markedly increased and expression of the anti-apoptotic regulator BCL-2 was decreased at 8 weeks post-immunization. This effect was reversed by CNTF or Reg-2 treatment. **(B,D)** (a) *P* < 0.05 vs. the normal control; (b) *P* < 0.05 vs. vehicle-treated EAE rats.

Western blotting showed that, at 8 weeks post-immunization in vehicle-treated rats, expression of the proapoptotic regulator BAX was clearly upregulated while expression of the antiapoptotic regulator BCL-2 was markedly downregulated (Figure 8). This phenomenon was reversed by CNTF or Reg-2 treatment.

**Figure 9:**
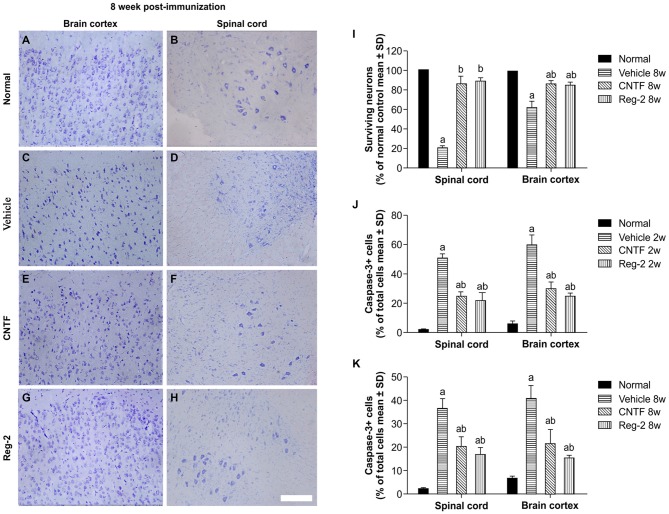
**CNTF treatment and Reg-2 treatment reduce apoptosis and neuronal loss in the spinal cord and brain cortex. (A–H)** Visible neuronal loss was observed in the CNS of the vehicle-treated rats at 8 weeks post-immunization **(C–D)** relative to normal animals **(A–B)**. In CNTF- and Reg-2-treated EAE rats, a greater number of neurons was observed in the anterior horn of the spinal cord and in the motor cortex **(E–H)**. Nissl staining, scale bar = 100 mm. **(I)** The calculated number of surviving neurons at 8 weeks post-immunization (each value is presented as a percentage of the normal control). (a) *P* < 0.05 vs. normal control; (b) *P* < 0.05 vs. vehicle-treated EAE rats. **(J,K)** The number of caspase-3-labeled neurons at 8 weeks post-immunization. (a) *P* < 0.05 vs. normal control; (b) *P* < 0.05 vs. vehicle-treated EAE rats.

Visible neuronal loss and activated caspase-3 expression was also observed in the CNS of vehicle-treated rats relative to normal rats at 8 weeks post-immunization (Figure 9). In CNTF- and Reg-2-treated rats, a larger number of neurons were visualized in the anterior horn of the spinal cord and in the motor cortex (Figure 9). No significant differences were observed between CNTF- and Reg-2-treated rats (Figure 9).

## Discussion

In MS and EAE, myelin repair is generally insufficient despite the survival of some oligodendrocytes in demyelinating plaques (Stankoff et al., 2002). In the present model of rodent EAE, we noted the appearance of remyelination during the recovery stage at 8 weeks post-immunization, but remyelination alone was insufficient to produce functional recovery in vehicle-treated rats. Therefore, the identification of functionally significant therapeutic targets is an obstacle to MS treatment.

CNTF was originally identified as a survival factor in isolated neurons, and was later additionally identified as a factor for the differentiation, maturation, and survival of oligodendrocytes. CNTF-deficient mice are thus reported to demonstrate poor recovery from the acute phase of EAE due to extensive oligodendrocyte apoptosis (Linker et al., 2002). Moreover, a greater extent of myelin vacuolar dystrophy and axonal damage has been reported in CNTF-deficient mice (Linker et al., 2002). In the present study, sustained delivery of recombinant rat CNTF or Reg-2 was used to treat acute EAE. Both CNTF and Reg-2 exerted direct neuroprotective effects, alleviating demyelination, preventing axon loss, and reducing neuronal death. Both treatments also led to a delay in disease onset and clinical manifestation.

CNTF and Reg-2 treatments reduced the number of infiltrating T cells and activated macrophages including microglia during the early stages of EAE. Previous studies have shown that CNTF inhibits the endotoxin-mediated synthesis of pro-inflammatory mediators IL-1 and prostaglandin E_2_ in a receptor-dependent fashion (Shapiro et al., 1994). Additionally, transplantation of mesenchymal stem cells transfected with CNTF decreases serum TNF-α and IFN-γ and reduces the infiltration of inflammatory and CD3-positive cells into EAE lesions (Lu et al., 2009). Moreover, endogenous CNTF is thought to have a centrally mediated anti-inflammatory role for inhibiting the production of TNF-α and IL-6 (Meazza et al., 1997). Our data are consistent with these findings. CNTF and Reg-2 administration significantly downregulated the expression of pro-inflammatory cytokines TNF-α, IL-2, and IFN-γ, as well as inflammatory pathway-related transcription factors such as COX-2 and NF-κB. Conversely, these treatments increased the expression of anti-inflammatory cytokines IL-4 and TGF-β. These data suggest a possible immunosuppressive effect on CD4^+^ T helper1 (Th1) lymphocytes that favors the activity of CD4^+^ Th2 cells (Zhang et al., 2006; Garay et al., 2008).

At 8 weeks post-immunization (6 weeks after the cessation of treatment), inflammatory infiltration increased in CNTF- and Reg-2-treated rats; however, it appeared that the anti-inflammatory effects of CNTF and Reg-2 in the early stage had already improved the CNS microenvironment. As a result, more neurons were retained in the CNTF- and Reg-2-treated groups relative to the vehicle-treated group. Moreover, although CNTF and Reg-2 did not appear to have long-lasting effects on inflammation in the late stage, their neurotrophic effects persisted and appeared to alleviate demyelination, prevent axon loss, and reduce neuronal death. Thus even at 8 weeks post-immunization, improvements were observed in remyelination as well as axonal and neuronal survival. We speculated that at this stage, infiltrating lymphocytes were likely to be Th2-polarized cells. This hypothesis was confirmed by the observation of double-labeled CD4-positive and CCR3-positive cells in the CNS, which indicated the infiltration of Th2-polarized rather than Th1-polarized lymphocytes. These data suggest that CNTF treatment and Reg-2 treatment similarly retain the ability to alleviate demyelination, axonal loss, and neuronal death even after treatment cessation.

NF-κB is a transcription factor that is central to immunity and inflammation. This protein regulates the expression of a number of genes related to cytokines and apoptosis (Yin et al., 2012). TNF-α has an established role in oligodendrocyte apoptosis and myelin destruction in EAE (Linker et al., 2002). Inflammation-mediated damage and apoptosis have also been attributed to the overproduction of COX-2 (Song et al., 2014). Given that the expression of these pre-apoptotic factors was effectively reduced by CNTF and Reg-2 treatments, we anticipated the observation of increased anti-apoptotic BCL-2 and decreased pro-apoptotic BAX and caspase-3. Indeed, these findings are in accordance with the rescue of oligodendrocytes and neurons by direct neurotrophic effects and an improved cellular microenvironment (Yu et al., 2010; Malairaman et al., 2014).

Regenerative sprouting and axon growth are regulated by the intrinsic growth potential of the neuron as well as the inhibitory environment present in the myelinated adult CNS (Fitch and Silver, 2008). GAP-43, also known as neuromodulin, is a protein considered to be crucial for axonal regeneration (Jacobson et al., 1986). Neurons in a regenerative state show increased expression of GAP-43, while decreases in GAP-43 expression are thought to result from neuronal damage (Saal et al., 2016). Thus, changes in GAP-43 expression likely reflect the induction of a nerve regenerative program (Ceber et al., 2015). In our study, we observed that CNTF and Reg-2 treatments prolonged initial increases in GAP-43 expression induced by EAE induction. Thus supporting the expression of GAP-43 may have played a role in the ability of CNTF and Reg-2 to prevent axon degeneration and promote axon regeneration in EAE.

Previous research has revealed that purified Reg-2 has neurotrophic effects on subpopulations of motoneurons (Nishimune et al., 2000). As an obligatory intermediate in the survival signaling pathway mediated by CNTF (Emerich, 2004), Reg-2 is also itself a neurotrophic factor (Nishimune et al., 2000). Our studies confirm these observations: Reg-2 and CNTF produced similar inflammatory suppression, axon and myelin preservation, neuronal survival, and functional improvement in the acute phase of EAE. With a smaller molecular weight and improved penetration into the brain parenchyma, Reg-2 may be a useful substitute for CNTF and help to avoid the many adverse effects of direct CNTF application in EAE and MS (Kuhlmann et al., 2006).

## Author Contributions

SH designed the experiments and drafted the manuscript, HJ participated in study design and coordination, K-WT and FZ performed the experiments, and BW analyzed the data and revised the manuscript. All authors read and approved the final manuscript.

## Conflict of Interest Statement

The authors declare that the research was conducted in the absence of any commercial or financial relationships that could be construed as a potential conflict of interest.

## Supplementary Material

Supplementary Figure 1 **The effect of ciliary neurotrophic factor (CNTF) treatment and regeneration gene protein 2 (Reg-2) treatment on inflammatory cell infiltration.** Two weeks post-immunization, inflammatory cell infiltration was observed surrounding the blood vessels and in the CNS parenchymata of vehicle-treated rats **(B–C)** relative to normal control rats **(A)** the arrow in **(C)** shows perivascular cuffing. CNTF and Reg-2 treatments alleviated inflammatory cell infiltration **(D,E)**. However, at 8 weeks post-immunization **(F)**, 6 weeks after the cessation of treatment, inflammatory cell infiltration increased in CNTF- **(G)** and Reg-2-treated rats **(H)** relative to that observed at 2 weeks post-immunization. Nissl staining, Scale bar = 100 mm.
