# Supplementary figures and images for: Reg-2, A Downstream Signaling Protein in the Ciliary Neurotrophic Factor Survival Pathway, Alleviates Experimental Autoimmune Encephalomyelitis
Source: Front Neuroanat. 2016 May 9;10:50. doi: 10.3389/fnana.2016.00050 (PMC4860402; doi:10.3389/fnana.2016.00050)

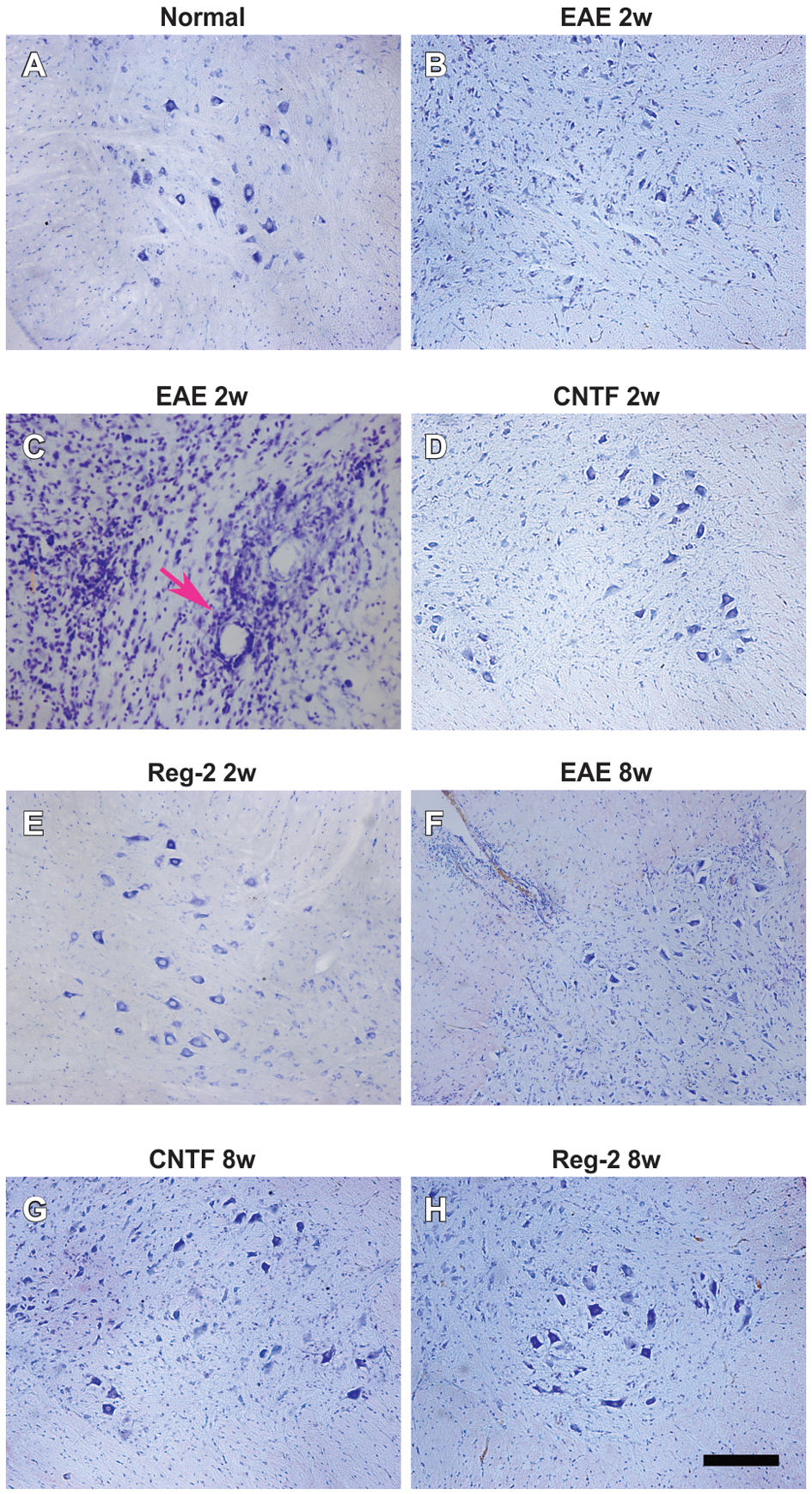

Supplement: Supplementary Figure 1 — The effect of ciliary neurotrophic factor (CNTF) treatment and regeneration gene protein 2 (Reg-2) treatment on inflammatory cell infiltration. Two weeks post-immunization, inflammatory cell infiltration was observed surrounding the blood vessels and in the CNS parenchymata of vehicle-treated rats (B–C) relative to normal control rats (A) the arrow in (C) shows perivascular cuffing. CNTF and Reg-2 treatments alleviated inflammatory cell infiltration (D,E). However, at 8 weeks post-immunization (F), 6 weeks after the cessation of treatment, inflammatory cell infiltration increased in CNTF- (G) and Reg-2-treated rats (H) relative to that observed at 2 weeks post-immunization. Nissl staining, Scale bar = 100 mm. [file Image_1.tif]
